# The Hemodynamic Mass Action of a Central Pattern Generator

**DOI:** 10.3389/fnins.2020.00038

**Published:** 2020-01-31

**Authors:** Mayra Moreno-Castillo, Roberto Meza, Jesús Romero-Vaca, Nayeli Huidobro, Abraham Méndez-Fernández, Jaime Martínez-Castillo, Pedro Mabil, Amira Flores, Elias Manjarrez

**Affiliations:** ^1^Instituto de Fisiología, Benemérita Universidad Autónoma de Puebla, Puebla, Mexico; ^2^Centro de Investigación en Micro y Nanotecnología, Universidad Veracruzana, Veracruz, Mexico

**Keywords:** central pattern generation, hemodynamics, BOLD, DC-photoplethysmography, fNIRS

## Abstract

The hemodynamic response is a neurovascular and metabolic process in which there is rapid delivery of blood flow to a neuronal tissue in response to neuronal activation. The functional magnetic resonance imaging (fMRI) and the functional near-infrared spectroscopy (fNIRS), for instance, are based on the physiological principles of such hemodynamic responses. Both techniques allow the mapping of active neuronal regions in which the neurovascular and metabolic events are occurring. However, although both techniques have revolutionized the neurosciences, they are mostly employed for neuroimaging of the human brain but not for the spinal cord during functional tasks. Moreover, little is known about other techniques measuring the hemodynamic response in the spinal cord. The purpose of the present study was to show for the first time that a simple optical system termed direct current photoplethysmography (DC-PPG) can be employed to detect hemodynamic responses of the spinal cord and the brainstem during the functional activation of the spinal central pattern generator (CPG). In particular, we positioned two DC-PPG systems directly on the brainstem and spinal cord during fictive scratching in the cat. The optical DC-PPG systems allowed the trial-by-trial recording of massive hemodynamic signals. We found that the “strength” of the flexor-plus-extensor motoneuron activities during motor episodes of fictive scratching was significantly correlated to the “strengths” of the brainstem and spinal DC-PPG signals. Because the DC-PPG was robustly detected in real-time, we claim that such a functional signal reflects the hemodynamic mass action of the brainstem and spinal cord associated with the CPG motor action. Our findings shed light on an unexplored hemodynamic observable of the spinal CPGs, providing a proof of concept that the DC-PPG can be used for the assessment of the integrity of the human CPGs.

## Introduction

The word plethysmograph comes from the Greek “plethismos” (becoming full or enlargement) and “graphos” (to write). This word is similar to the more known term “plethora,” meaning overabundance or the state of being full. In medicine, the word “plethysmograph” describes an instrument for registering variations in the volume of an organ, limb, or from the whole body. In the case of the limbs, the plethysmography is used for registering variations in the volume of arms and legs, and hence the variations in the amount of blood in these limbs. Plethysmographs were also used a long time ago to measure lung volume via the law of Boyle-Mariotte (Haldane and Priestley, 1905; Criée et al., 2011). In 1938, Hertzman introduced the term “photoelectric plethysmography,” also known as “Photoplethysmography” (PPG), to estimate the blood supply of skin areas (Hertzman, 1938). This concept born from a previous study by Hertzman and Spealman (1937), in which they reported the photo-electrical pulsatile nature of blood supply from the fingertip.

The PPG is a simple optical method that employs a light source to illuminate the tissue and a photodetector that detects the reflected or transmitted light. A PPG signal can be separated into an oscillating (AC) and a steady-state (DC) component. The amplitude of both components is dependent upon the structure and flow in the microvascular bed, oxygen consumption, and other unknown factors (Kamal et al., 1989). From its implementation, this technique has commonly been employed in non-invasive devices, from single spot monitoring to more advanced imaging PPG (IPPG) and non-contact IPPG (Sun and Thakor, 2015), mainly to detect the AC-PPG component. The IPPG uses fast digital cameras and advanced software (for instance, see Liu et al., 2018). The PPG and contact/non-contact IPPG are widely employed for clinical purposes to obtain non-invasive recordings of blood pressure, oxygen saturation, respiration, and heart rate (for review, see Allen, 2007; Sun and Thakor, 2015). Recently, the PPG has been employed for quantitative assessment of hypertension (Liang et al., 2018), non-contact neonatal monitoring (Cobos-Torres et al., 2018), sympathetic nerve activity during cold stress (Budidha and Kyriacou, 2019), among a great variety of other applications.

In 2006, Phillips and colleagues developed an optical fiber PPG system to detect by reflectance “AC” PPG signals from the rat spinal cord (AC-PPG). They introduced such a system as a proof of concept that a similar system could be employed in the future in human subjects to examine mechanisms of spinal cord injury (Phillips et al., 2006). In a second report, Phillips et al. (2009) employed the same system to investigate whether the pulsation of small arteries in the rat spinal cord occurred before, during, and after compressive loads applied to the spinal cord. They found that the pulsatile AC-PPG signal amplitudes were reduced by compressive loads, with a reduction that persisted for at least 5 min after the compression ended. These authors suggested that ischemia may occur during compressive injury of the spinal cord. In a subsequent study, Phillips et al. (2013) employed the same AC-PPG fiber-optic probe to assess the regional perfusion of the spinal cord in anesthetized adult rats. They found a considerable inter-site and inter-subject variability in the AC-PPG signal compared to the Doppler flowmetry. They concluded that the AC-PPG could be employed to investigate pathological mechanisms of spinal cord injury. Recently, Cibert-Goton et al. (2015) employed the same technique of AC-PPG in the rat spinal cord to show that the spinal root avulsion does not significantly alter blood flow or tissue oxygen levels, suggesting that ischemia may play a less prominent role in avulsion injury-induced pain than previously thought. In another study, Phillips et al. (2008) reported a similar fiber-optic cerebral oximetry system with AC–DC capabilities to be used in the brain tissue of patients recovering from neurosurgery or head injury.

These findings of AC-PPG signals from the rat spinal cord, and the fact that it is possible to obtain direct current photoplethysmography (DC-PPG) signals from the brain (Phillips et al., 2008), motivated our laboratory to examine the characteristics of the “functional DC-PPG” signals in the *in vivo* brainstem and spinal cord during a fictive motor task. Therefore, our study aimed to examine whether the functional DC-PPG is useful to detect the physiological activity of the brainstem and spinal cord in real-time during a scratching motor task in decerebrate cats. We suggest that the functional DC-PPG complements the AC-PPG, and it could also be employed for the real-time assessment of the central pattern generation function.

## Materials and Methods

### Animal Preparation

We made an effort to reduce the number of cats used in this study, in agreement with the guidelines contained in the Mexican regulations (NOM-062-ZOO-1999) for the care and use of laboratory animals. With this idea, we performed experiments in seven adult cats (2.3–3.7 kg). Our experimental procedures are similar to those employed in previous reports from our group (Cuellar et al., 2009, 2018; Tapia et al., 2013; Meza et al., 2019). The animals were handled with care and introduced in a comfortable anesthesia-induction-box at a temperature of 22–29°C and 40–70% relative humidity. During surgery, we employed isoflurane at 2% to maintain deep anesthesia, and we applied atropine and dexamethasone (0.05 and 2 mg/kg, respectively). The ethics committee (CICUAL-Proyecto-00489) from the Benemérita Universidad Autónoma de Puebla approved our experimental protocol. We followed the guidelines contained in the Mexican regulations (NOM-062-ZOO-1999) for the care and use of laboratory animals and the corresponding National Institutes of Health Guide. We verified the level of anesthesia by testing for the lack of withdrawal reflexes and muscle tone and by the monitoring of arterial blood pressure from the carotid artery. During all the experiments, we administered a mix of bicarbonate (100 mm) and glucose (5%) solution throughout the radial vein at a rate of 5 ml/h.

For electrophysiological recording, we dissected the bilateral tibialis anterior (TA) and the medial gastrocnemius (MG) nerves. For optical recording, the lumbosacral spinal cord segments were exposed, and the dura mater was removed. We mounted the animal on a stereotaxic apparatus. The skin around the exposed tissues was used to form pools, which were filled with mineral oil (after placement of the electrodes and optic systems) and maintained at a constant temperature (37°C). We made a decerebration, which consisted of a mechanical precollicular–postmamillary transection with the complete removal of all tissue rostral to the transection and both cerebral hemispheres. To avoid bleeding, we applied SURGICEL^®^ Absorbable Hemostat on the exposed neural tissues. The empty cavity was filled with Agar-Agar. The anesthesia was discontinued 5 min after the decerebration verifying that the respiration, blood, pressure, and heartbeat were stable. We noted that even after the discontinuation of the anesthesia, the animals did not exhibit any sign of discomfort because they were correctly decerebrate, and all centers of conscious pain were carefully removed. To maintain blood pressure between 80 and 120 mmHg, we administered dextran and saline solutions as necessary. At the end of all surgical procedures, including the decerebration, we administered pancuronium bromide (Pavulon; Organon), and then the animals were artificially ventilated. We applied *d*-tubocurarine (0.1%) on the surface of the C1–C2 segments using a piece of cotton impregnated with it. Scratching was produced by a brief (about 1 s) mechanical stimulation of scratch reflex receptive fields located on the left pinna. At the end of the experiments, each animal was euthanized with an overdose of pentobarbital, and the spinal cord was recovered for histological analysis.

### Implementation of Our Functional DC-PPG System to the Spinal Cord

**FIGURE 1:**
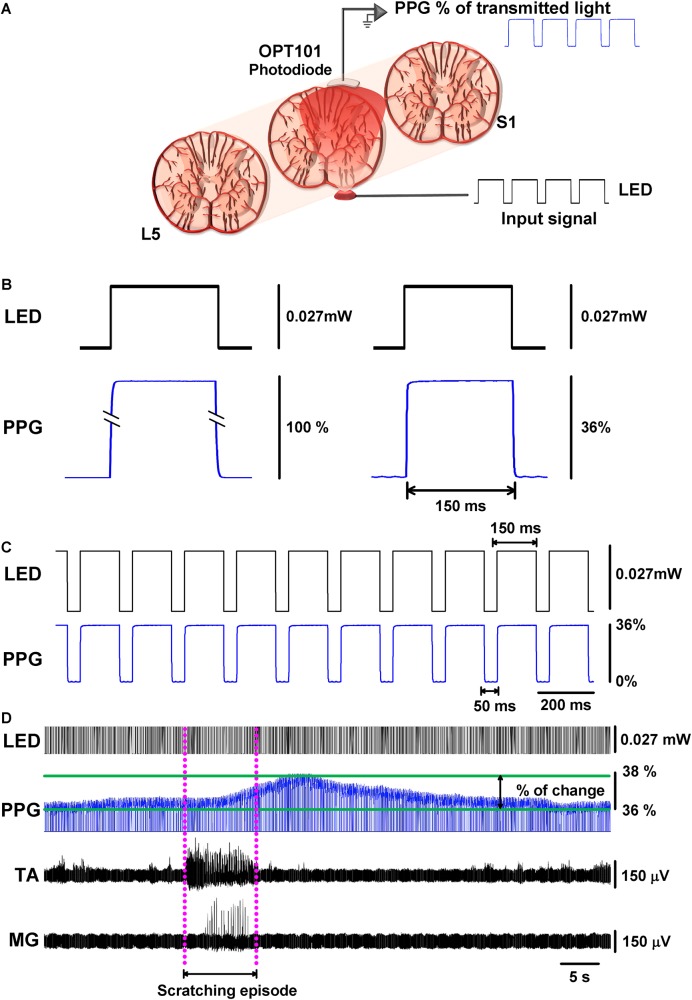
Functional DC-PPG applied to the cat spinal cord during an episode of motor activity. **(A)** Scheme of the experimental arrangement, illustrating a red LED below the L6 lumbar spinal segment, and a Texas Instruments OPT-101 photodiode on the spinal cord. The dura mater was removed to allow that both devices could touch, but not press the spinal cord. **(B)** Comparison between the DC-PPG response to LED illumination, with and without the spinal cord. Without any tissue in between, the photodiode detects 100% of the red light (left panel). Note how a LED pulse produces attenuation from 100% to about 36% of the red light when it crosses the spinal cord (right panel). **(C)** Upper panel, a train of red-LED pulses of 150 ms with a dark interval of 50 ms and an amplitude of 0.027 mW of light intensity applied throughout the spinal cord, as illustrated in panel **(A)**. **(C)** Lower panel, the OPT-101 photodiode response in the spinal cord (i.e., a zoom in of the pulsed elements of the functional DC-PPG signal); note that the spinal cord tissue attenuated the light to 36%. **(D)** Typical recording in real-time of the functional DC-PPG signal in the spinal cord elicited during an episode of motor action (fictive scratching). Note: how the tibialis anterior (TA) and medial gastrocnemius (MG) nerve activities precede by few seconds the maximal peak response of the functional DC-PPG signal detected with the OPT-101 photodiode. The dashed pink lines illustrate the length of the fictive scratching episode (i.e., the motor activity episode).

The functional DC-PPG system for the spinal cord consisted of two elements: (1) a miniature red LED (with peak emission wavelength at 660 nm) placed below the spinal cord and above the remaining dura mater and vertebral body and (2) a Texas Instruments OPT-101 photodiode placed directly above the spinal cord, on its left side for left scratching (Figure 1A). The signal obtained from the functional DC-PPG system was expressed as a percentage of the transmitted light through the tissue.

The device was held in place using a micromanipulator and a series of holders and posts attached to the stereotaxic frame where the cat was placed. We take care to ensure that the device touched the surface of the spinal cord without pressing it. The metal components of both devices were adequately insulated. Once the functional DC-PPG system was placed, a small pool of black agar-agar was poured on and around the optical devices. The black color insured insulation from possible light pollution from the otherwise darkened room. The LED was driven by a MASTER-8 stimulator (AMPI, Israel), while the OPT-101 photodiode was directly connected to an input channel of a DIGIDATA (Molecular Devices) data acquisition system (in a free-run mode at a 10 kHz sampling-rate, without filtering to allow a continuous DC-recording without averaging).

### LED-Illumination Protocol and Recording

Our LED-illumination protocol consisted of pulses of 150 ms of red light (peak emission wavelength at 660 nm) with 50 ms intervals of darkness (Figures 1B,C). We used an optical power meter PM100D-Thorlabs to measure the light intensity for different input voltages to the red LED delivered by the MASTER 8. We adjusted the amplitude of light pulses to 0.0027 mW (3.5 V from the MASTER 8). We simultaneously recorded the electroneurographic activity of the TA and MG nerves, as well as the functional DC-PPG signal from the lumbar L6 spinal cord. The electroneurographic activity was amplified ×5000 with Grass-Astromed (P511) amplifiers (0.05 Hz to 30 kHz band-pass).

### Experimental Paradigm

The experimental paradigm consisted of the simultaneous electroneurographic recording of the TA and MG nerves, and the functional DC-PPG signals during scratching episodes elicited by brief mechanical stimulation (about 1 s) of the left pinna. To avoid fatigue, we elicited each scratching episode at least 30–60 s after the ending of a previous scratching episode.

**FIGURE 2:**
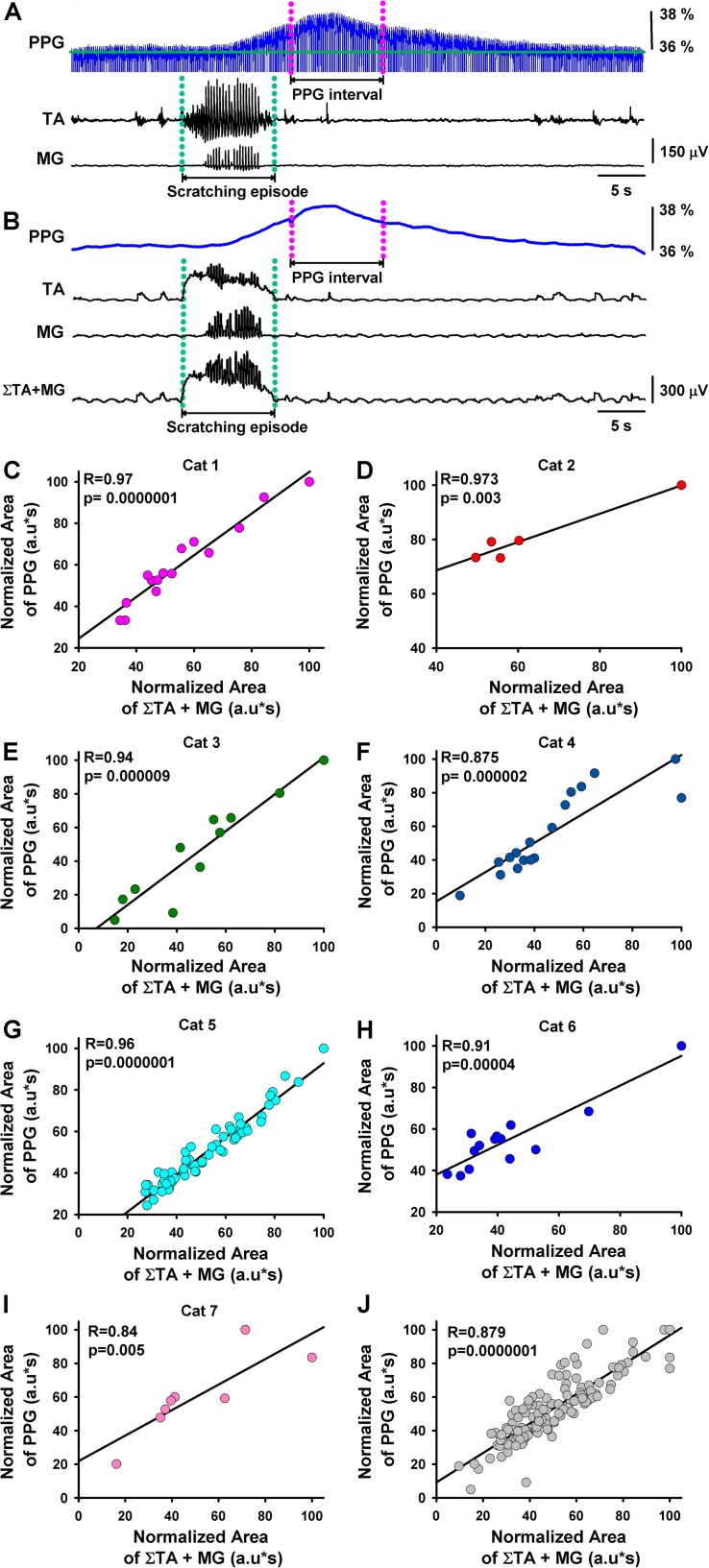
Correlation between the functional DC-PPG in the spinal cord versus the summated motor response during episodes of motor action. **(A)** The same as Figure 1D, but for another episode of fictive scratching. A functional DC-PPG interval was defined as follows: (1) The magenta lines delimit the duration of the functional DC-PPG interval equal to the duration of the scratching episode, indicated with green lines. (2) We positioned the functional DC-PPG interval around its maximal peak amplitude. **(B)** The same as in panel **(A)**, but after the functional DC-PPG signal was lowpass filtered, and the TA and MG electroneurograms were integrated and rectified. The lower panel in panel **(B)** shows a sum of the integrated and rectified TA and MG signals. **(C–I)** Results obtained from normalized data of seven cats. The graph shows the “normalized area below the summated TA and MG motor responses” versus the “normalized area below the functional DC-PPG signal” in the spinal cord during the fictive scratching episodes. **(J)** Superimposed graphs illustrated in Panels **(C–I)**, Pearson’s correlation with 140 degrees of freedom (df). The coefficient (*R*) and *p*-value are indicated. Note that all correlations are statistically significant *p* = 0.005 in one case and *p* < 0.0001 for all the other cases.

**FIGURE 3:**
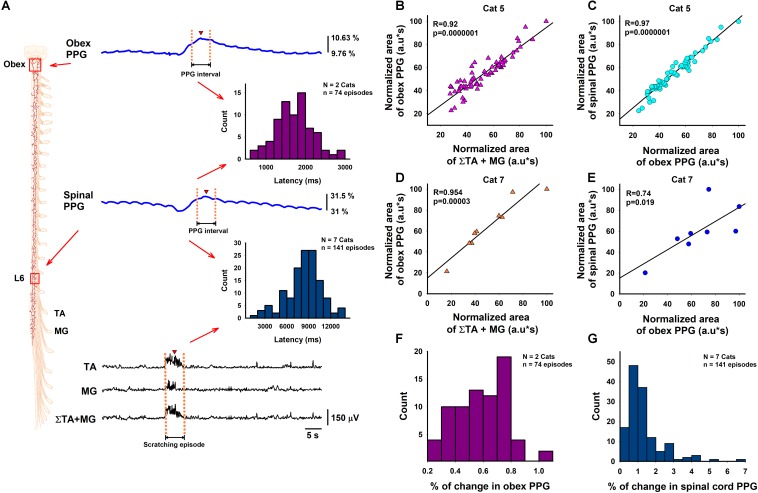
Correlations between the functional DC-PPG in the brainstem and the spinal cord versus the summated motor response during episodes of motor action. **(A)** Diagram of the experimental arrangement. Two Texas Instruments OPT-101 photodiodes were positioned on the brainstem and spinal cord to obtain simultaneous recordings of the functional DC-PPG signals (blue traces) during the motor action (black traces). The magenta histogram illustrates the latencies between the maximal points (red triangles) of the DC-PPG signal in the obex and the DC-PPG signal in the spinal cord. The blue histogram illustrates the latencies between the maximal point (red triangle) of the DC-PPG signal in the spinal cord and the mean interval of the summated TA and MG motor responses (red triangle). **(B)** Normalized data of the correlation between the obex PPG signal and the electroneurographic TA + MG signal. **(C)** Normalized data of the correlation between the spinal PPG signal and the obex PPG signal. **(D,E)** The same as panels (**B,C**), but for another animal. **(F,G)** Histograms of the percentage of change of the obex and spinal PPG signals for all the animals, as indicated (see also Table 1).

We performed two different types of experiments. The first type of experiment (Figure 2) consisted of the simultaneous recording of the DC-PPG signal in the spinal cord and the electroneurographic activity in the TA and MG nerves. The second type of experiment (Figure 3) consisted of the simultaneous recording of the DC-PPG signals in the brainstem and the spinal cord, plus the electroneurographic activity in the TA and MG nerves.

### Statistical Analysis

We performed several non-parametric pairwise Signed-Rank Tests to examine the statistical significance for the difference between the “DC-PPG before scratching” versus “DC-PPG after scratching” in the spinal cord and the brainstem, under the null hypothesis that the differences between conditions were zero. All effects are reported as significant if *p* < 0.05. One-tailed probability was considered for significance. Furthermore, a Spearman’s rank correlation method was used to test for significant correlations between the normalized area of the DC-PPG signal and the normalized area of the TA and MG electroneurographic activity. The correlation coefficients were calculated, and all correlations are reported as significant if *p <* 0.05.

## Results

### First Type of Experiment: Implementation of the Functional DC-PPG System to the Cat Spinal Cord During a Motor Task

Because we measured the hemodynamic activity of the spinal cord during a motor task, we termed it as “functional DC-PPG.” We show in Figure 1A the experimental arrangement. The left panel of Figure 1B shows that the LED illumination intensity of 0.027 mW produced 100% of the transmitted light when it is positioned directly on the photodiode without the spinal cord. The right panel of Figure 1B (see also Figure 1C) shows that the same LED illumination intensity of 0.027 mW produced a spinal PPG signal with 36% of the transmitted light when it travels through the spinal cord tissue. The relatively low percentage (36% in the spinal PPG) was expected due to the nature of this tissue (Jaques, 2013; Filatova et al., 2017). An important caveat is that our measurements were obtained on live tissue, which means that the light beam had to travel through 5–6 mm of the spinal cord with a continually fluctuating blood volume. Figure 1D shows a representative recording of the functional DC-PPG signal of the spinal cord (namely also PPG, or spinal PPG) during a fictive scratching episode. As expected, the activities of the TA and MG nerves during scratching were followed by a functional DC-PPG signal in the lumbar spinal cord. In Figure 1D, the dashed pink lines illustrate the onset and end of the motor episode. Similar recordings as those illustrated in Figure 1, were obtained in all the cats and for all the scratching episodes.

If we assume that the observed DC-shift in the functional DC-PPG signal of the spinal cord is related to a change in blood volume and flow of the spinal cord during scratching, then we could hypothesize that the magnitude in the activity of the TA and MG nerves could be correlated with such DC-shift in the functional DC-PPG signal. We examined this hypothesis with quantitative analysis for all the scratching episodes in seven cats. Such analysis is explained in Figures 2A,B.

Figure 2A illustrates with green dashed lines the time window of a scratching episode and with pink dashed lines a window of the same duration around the maximal-amplitude point of the functional DC-PPG signal of the spinal cord. Figure 2B illustrates the same as Figure 2A but with the filtering of the functional DC-PPG signal and rectification and integration of the TA and MG nerve activity, and the sum of both signals (TA plus MG). It is relevant to highlight that in our analysis, we always assumed a “time window of the scratching episode” equal to the “time window of the functional DC-PPG signal in the spinal cord.”

After we obtained the sum of the rectified and integrated TA and MG signals, we calculated its area. We also calculated the area below the functional DC-PPG signal of the spinal cord in the time window explained above. We made these calculations for 141 scratching episodes in seven cats. The results are illustrated in Figures 2C–I. Each plot shows the correlation between normalized data of the area of the sum of the rectified and integrated TA and MG signals versus the area of the functional DC-PPG signal in the same time window. We obtained Pearson’s correlation coefficients of 0.97, 0.97, 0.94, 0.87, 0.96, 0.91, and 0.84 for all the graphs, with a statistical significance of *p* ≤ 0.005 or *p* < 0.0001. Figure 2J shows pooled data for all the animals. The Spearman’s rank correlation method was used to test for significant correlations between the normalized area of the DC-PPG signal and the normalized area of the TA and MG electroneurographic activity. We obtained a *p* < 0.0001 with 140 degrees of freedom and a correlation coefficient of 0.87. This statistical analysis shows the reproducibility of our results. The blue histogram in Figure 3A illustrates the mean latency between the rectified and integrated TA + MG signals (red triangle) versus the functional DC-PPG signal of the spinal cord (red triangle) for these seven animals in 141 scratching episodes (mean latency = 8.3 ± 2.4 s, blue histogram).

### Second Type of Experiment: Implementation of the Functional DC-PPG System to the Cat Brainstem During a Motor Task

We also examined the correlation between normalized data of the rectified and integrated TA + MG signals versus the functional DC-PPG signal in the brainstem for two animals in 74 scratching episodes. Figures 3B,D show such correlations obtained from these two animals (Pearson’s correlation coefficients of 0.92 and 0.95, *p* < 0.001). We also analyzed the correlation between the areas of the PPG signal recorded in the obex versus the PPG signal recorded in the spinal cord (see Figures 3C,E). The correlation between both PPG signals was also highly significant, with Pearson’s correlation coefficients of 0.97 and 0.74, and *p* < 0.01. The magenta histogram in Figure 3A illustrates the mean latency between the functional DC-PPG signal of the brainstem (red triangle) versus the functional DC-PPG signal of the spinal cord (red triangle) for these two animals in 74 scratching episodes (mean latency = 1.7 ± 0.4 s). As expected, this latency between both DC-PPG signals is similar to the latency between the obex slow potential and the spinal cord DC-shift associated with the scratching episodes (see Tapia et al., 2013). All relevant data is contained within the manuscript.

Table 1 shows the number of scratching episodes analyzed, as well as the mean and median of the percentage of change in the DC-PPG signal (i.e., the % of change between the green lines in Figure 1D) for all the animals. The % of change in the DC-PPG signal compares the “DC-PPG before scratching” versus the maximal “DC-PPG after scratching.” The *p*-values were obtained from a pairwise Signed Rank test. Note the number of scratching episodes per animal was from 8 to 65. In fact, our results were obtained from the analysis of a total number of 141 scratching episodes in seven cats for the first type of experiment, and a total number of 74 scratching episodes in two cats for the second type of experiment (see also Figures 3F,G). Note that in all of these scratching episodes, we obtained a statistically significant “% of change” in the DC-PPG signal (*p* < 0.05). This statistical analysis shows the reproducibility of our results.

**TABLE 1:** Statistical significance of normalized amplitude of DC-PPG signal.

		**First type of experiment (spinal cord and nerves)**							**Second type of experiment (brainstem, spinal cord, and nerves)**		**Global**
				
		**Cat 1**	**Cat 2**	**Cat 3**	**Cat 4**	**Cat 5**	**Cat 6**	**Cat 7**	**Cat 5**	**Cat 7**	
Count	Number of Scratching episodes	16	8	11	14	65	18	9	65	9	215
Mean	Percentage of change	9.88 ± 3.7	0.4 ± 0.14	2.19 ± 1.89	2.67 ± 1.33	1.2 ± 5.55	2.51 ± 0.59	5.19 ± 3.23	4.66 ± 1.26	2.87 ± 0.51	3.35 ± 4.14
Median	Percentage of change	8.34	0.35	0.89	2.45	1.91	2.31	4.43	4.82	3.01	2.85
*T*		0	0	0	0	0	0	0	0	0	1
*z*		−3.54	−2.52	−2.94	−3.29	−6.59	−3.72	−2.67	−7.02	−2.75	−12.48
*r*		−0.89	−0.89	−0.88	−0.87	−0.82	−0.88	−0.89	−0.87	−0.92	−0.85
*p*		0.0002	0.006	0.0015	0.005	0.0000001	0.00009	0.004	0.0000001	0.003	0.0000001

## Discussion

We found that the brainstem and spinal DC shifts in the functional DC-PPG signals are associated with the magnitude of the motor output during a fictive motor task in the cat (to our knowledge, this is the first report of such finding). Although there is the limitation that our current application is invasive and a specific cause-effect explanation is beyond the scope of this study, this observable effect was quantified in order to obtain the first approach to this significant correlation. However, we suggest that relevant hemodynamic processes may be occurring during such functional DC-PPG signals. Our results represent a proof-of-concept that a similar functional-DC-PPG system, based on the non-invasive functional near-infrared spectroscopy (fNIRS) technique, could be employed in the human spinal cord and brainstem to access the integrity of neuronal circuits in such regions during motor tasks involving DC-shift changes in their PPG signals.

Proof-of-concept tests have been reported before (e.g., Phillips et al., 2006, 2009, 2013; Cibert-Goton et al., 2015) but for pulsatile AC-PPG signals that were obtained in the rat spinal cord by a light reflection method. In such studies, the aim was the investigation of the possible use of the AC-PPG as a diagnostic tool for the evolution of spinal compressive injuries. However, the purpose of the present work was to look into the viability of the functional DC-PPG to obtain reliable optical signals of the spinal cord and brainstem correlated to the spinal motoneuron output during a motor task. Because two DC shifts in the functional DC-PPG signals were detected in the lumbar spinal cord and brainstem during scratching, it is possible that such DC shifts indirectly reflect the concerted activity of brainstem-spinal central pattern generator (CPG) networks and motoneurons.

We observed a time lag of about 8.3 ± 2.4 s between the electroneurographic activity of the TA + MG nerves and the functional DC-PPG recorded in the spinal cord. This finding suggests that the functional DC-PPG represents a hemodynamic response associated with the metabolic demands of the motor task in the spinal cord. This hemodynamic response may involve the activation of various cell types, like astrocytes, endothelial cells of blood vessels, and pericytes, among other cells in the lumbar spinal cord. It is also plausible that during such hemodynamic response these cells can take control of the dilatation or constriction of the spinal vessels intermixed in the neuronal tissue, thus dictating the amount of oxygen and glucose that can reach the active neuronal-ensembles (Chen et al., 2012; Sedwick, 2012). We suggest that such a cascade of hemodynamic events last about 8.3 ± 2.4 s in our experiments, thus explaining the latency between the TA + MG motor output and the functional DC-PPG signal in the spinal cord. However, such mechanisms must be demonstrated in future experiments. One point supporting our suggestions is the similitude in the delay observed for the hemodynamic response in the functional magnetic resonance imaging (fMRI). In the brain, the fMRI measures the subsequent demand for oxygenated blood that follows about 6 s after the neuronal electrical response (Liao et al., 2002). Such parallelism in the time lag between our functional DC-PPG in the spinal cord (8.3 ± 2.4 s) and the fMRI (around 6 s) is notable, and it should be examined in detail in future experiments. In this context, our device, and methods also provide a proof-of-concept for the viability of a functional DC-PPG system for functional imaging, which could be as powerful as the fMRI.

Our findings are consistent with those obtained in the human brain with the simultaneous use of the fNIRS and the fMRI (for review see Steinbrink et al., 2006; Scarapicchia et al., 2017). There is compelling evidence that the changes in the BOLD fMRI signal are related to changes in deoxy-Hb, total-Hb, and regional cerebral blood volume during a variety of sensory, motor, cognitive tasks and resting states (Sakatani et al., 2007; Cui et al., 2011; Duan et al., 2012; Quaresima et al., 2012; Sasai et al., 2012; Tong et al., 2012; Sato et al., 2013; Yuan and Ye, 2013; Fabiani et al., 2014; Hocke et al., 2015; Noah et al., 2015; Anwar et al., 2016; Vannasing et al., 2016). Our findings are also supported by studies in mice with the simultaneous recording of fluorescent-based calcium recordings and BOLD fMRI signals (Schlegel et al., 2018). In this context, our findings suggest that a variant of the fNIRS in DC mode could also be developed to explore functional optical signals related to specific changes in deoxy-Hb, oxy-Hb, total-Hb, and/or regional cerebral blood volume contributing to the hemodynamic response of the spinal cord and the brainstem. Recent studies employing functional ultrasound imaging of spinal cord hemodynamic responses to epidural stimulation (Song et al., 2019) provide support to such possibility.

We conclude that it is possible to record in real-time a reliable functional-DC-PPG hemodynamic signal in the spinal cord and brainstem during a motor task. This suggests that the functional-DC-PPG is a hemodynamic method that could be employed for the assessment of the CPG integrity of the spinal cord and brainstem.

## Data Availability Statement

The raw data supporting the conclusions of this article will be made available by the authors, without undue reservation, to any qualified researcher.

## Ethics Statement

The animal study was reviewed and approved by the Ethics Committee of Benemérita Universidad Autónoma de Puebla (CICUAL-Proyecto-00489).

## Author Contributions

EM and AF conceived and designed the experiments, and wrote the manuscript. MM-C, RM, JR-V, AF, NH, and EM performed the experiments. MM-C, AM-F, JM-C, NH, PM, AF, and EM performed the analysis. All authors revised and approved the manuscript.

## Conflict of Interest

The authors declare that the research was conducted in the absence of any commercial or financial relationships that could be construed as a potential conflict of interest.
